# Supplementary material for: Effects of Stocking Density and Pre-Slaughter Handling on the Fillet Quality of Largemouth Bass (Micropterus salmoides): Implications for Fish Welfare
Source: Foods. 2024 May 10;13(10):1477. doi: 10.3390/foods13101477 (PMC11120225; doi:10.3390/foods13101477)
Supplement: Supplementary file 1 [file foods-13-01477-s001.zip › foods-2979777-supplementary.pdf]

Table S1. Three-Way ANOVA p-value table for the data variables.

**Effect of (p-value)→** **Variable ↓**	**Slaughtering method**	**Stocking density**	**Storage time**	**Slaughtering method X Stocking density**	**Slaughtering method X Storage time**	**Stocking density X Storage time**	**Slaughtering method X Stocking density X Storage time**
**Rigor index**	7.00E-03	1.19E-01	5.23E-31	3.68E-05	1.56E-22	1.90E-16	6.77E-09
**pH**	4.40E-02	1.02E-04	1.60E-10	4.20E-02	5.40E-10	4.94E-04	6.50E-02
**Hardness**	1.25E-04	9.00E-06	3.85E-04	4.81E-04	3.00E-02	1.00E-03	1.89E-05
**Lipid oxidation (TBARS)**	4.81E-04	1.90E-04	4.25E-07	5.30E-02	9.81E-04	4.40E-02	1.53E-01

Significance interactions <0.05, <0.01, <0.001, <0.0001 are indicated as the green colour cells.
